# Chemical Composition and Synergistic Antifungal Properties of Essential Oils from Ginger, Kaffir Lime and Galangal Against *Trichophyton interdigitale*, *Aspergillus niger* and *Candida albicans*

**DOI:** 10.3390/molecules31213951

**Published:** 2026-11-02

**Authors:** Weerasak Samee, Pavornwus Sununtanapaisal, Kwanchanok Vimuktanon, Sarin Tadtong

**Affiliations:** Faculty of Pharmacy, Srinakharinwirot University, Nakhon Nayok 26120, Thailand; weerasak@g.swu.ac.th (W.S.);

**Keywords:** essential oil, hydrodistillation, antifungal, synergism, gas chromatography

## Abstract

This study developed blended essential-oil formulations with antifungal activity against superficial fungal pathogens, including *Trichophyton interdigitale*, *Candida albicans*, and *Aspergillus niger*. Essential oils were obtained by hydrodistillation from ginger (*Zingiber officinale* Roscoe), galangal (*Alpinia galanga* (L.) Willd.), and kaffir lime (*Citrus hystrix* DC.), with yields of 0.09%, 0.16%, and 0.37% (*w*/*w*), respectively. Individual antifungal activities were assessed using the broth dilution method. Galangal and kaffir lime oils exhibited the greatest activity against *T. interdigitale* (minimum inhibitory concentration [MIC], 0.125% *v*/*v*), whereas ginger oil was the most effective against *C. albicans* (MIC, 0.25% *v*/*v*). Kaffir lime oil showed the strongest activity against *A. niger* (MIC, 0.125% *v*/*v*). Oil blends were subsequently prepared at ratios of 1:3, 1:1, and 3:1. The most active combinations were ginger–kaffir lime (3:1) against *T. interdigitale* (MIC, 0.00195% *v*/*v*), ginger–kaffir lime (1:3) against *C. albicans* (MIC, 0.0078% *v*/*v*), and galangal–ginger (1:1) against *A. niger* (MIC, 0.0625% *v*/*v*). Mineral oil was the most suitable carrier. The final formulations contained 2.5% blended oil for *T. interdigitale* and *C. albicans* and 10% for *A. niger*. These preliminary findings indicate that blended ginger, galangal, and kaffir lime essential oils may have potential for developing topical antifungal formulations against superficial fungal infections.

## 1. Introduction

Infectious diseases arise when pathogenic microorganisms invade the human body, then undergo complex interactions with the host and cause pathological changes. These causative agents include bacteria, fungi, viruses, and parasites [1]. Moreover, microorganisms that are part of the normal microbiota and typically remain harmless may become pathogenic under adverse conditions. Factors such as physiological imbalance, impaired immune function, and insufficient rest can encourage their excessive growth, ultimately contributing to the development of disease [2,3,4].

Thailand’s hot and humid climate, characterized by an average annual temperature of approximately 28 °C [5], creates favorable conditions for fungal proliferation [6]. The anticipated increase in rainfall may further enhance environmental humidity. In addition, several common occupations, including farming, fishing, vending, and cleaning, involve frequent exposure to water, potentially increasing the risk and prevalence of superficial fungal infections [7].

Three prevalent types of superficial fungal infection include athlete’s foot, commonly caused by *Trichophyton interdigitale* [8]; cutaneous candidiasis or Candida-associated dermatitis, caused by *Candida albicans* [9]; and external ear fungal infections, which are frequently linked to *Aspergillus species* [10]. Although conventional antifungal drugs are widely used, herbal essential oils have attracted increasing attention as potential alternative treatments. Numerous studies have demonstrated the antifungal properties of natural products, particularly medicinal plants, and several Thai medicinal plants have shown significant antifungal activity [11,12,13]. Herbal remedies have also been traditionally used to treat fungal infections and are generally considered accessible, affordable, and potentially safe when used appropriately.

Previous research has indicated that essential oils derived from ginger rhizomes (*Zingiber officinale* Roscoe) and galangal rhizomes (*Alpinia galanga* (L.) Willd.), both members of the Zingiberaceae family, as well as kaffir lime leaf (*Citrus hystrix* DC.) of the Rutaceae family, possess antifungal properties. These effects are associated with their bioactive chemical constituents [11,14]. Ginger essential oil contains zingiberene, geranial, α-curcumene, β-bisabolene, and β-sesquiphellandrene [11]. Galangal oil contains 1,8-cineole as a major component with antifungal properties [15], whereas kaffir lime leaf oil contains limonene, citronellal, and β-pinene [11,14,16], all of which have been associated with antifungal activity [11,16].

Accordingly, this study evaluated the antifungal activities of essential oils extracted from ginger rhizome, galangal rhizome, and kaffir lime leaf, both individually and in combination. Their efficacy was assessed against three fungal species: *T. interdigitale*, *C. albicans*, and *A. niger*. The study further sought to determine the optimal blending ratios for maximizing antifungal activity. In addition, incorporating these essential oils into an oil-based formulation may enhance their penetration into the skin, potentially improving their therapeutic effectiveness in treating superficial fungal infections.

## 2. Results

### 2.1. Extraction Yield of Essential Oil

Hydrodistillation of ginger rhizome, kaffir lime leaf, and galangal rhizome resulted in varying essential oil yields. Kaffir lime leaf produced the highest yield at 0.37% *w*/*w*, followed by galangal rhizome at 0.16% *w*/*w* and ginger rhizome at 0.09% *w*/*w*. The results are summarized in Table 1.

### 2.2. Chemical Composition of Essential Oils

**Figure 1:**
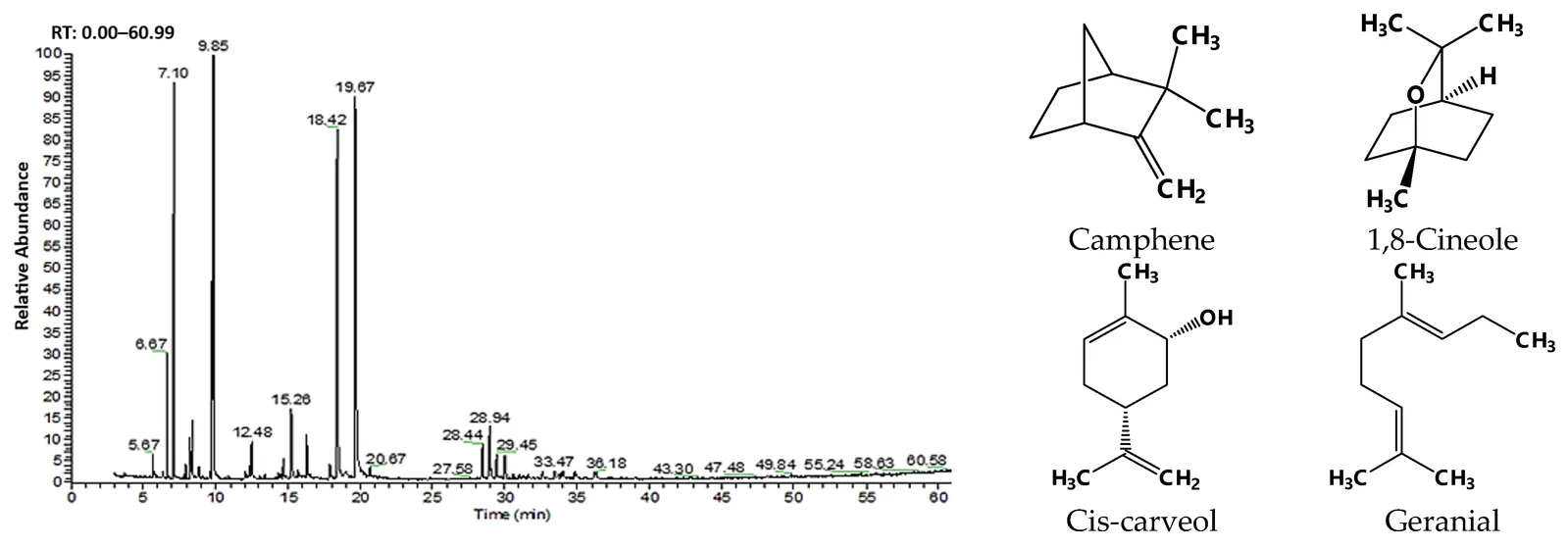
GC-MS chromatograms of essential oil extracted from ginger rhizome.

**Figure 2:**
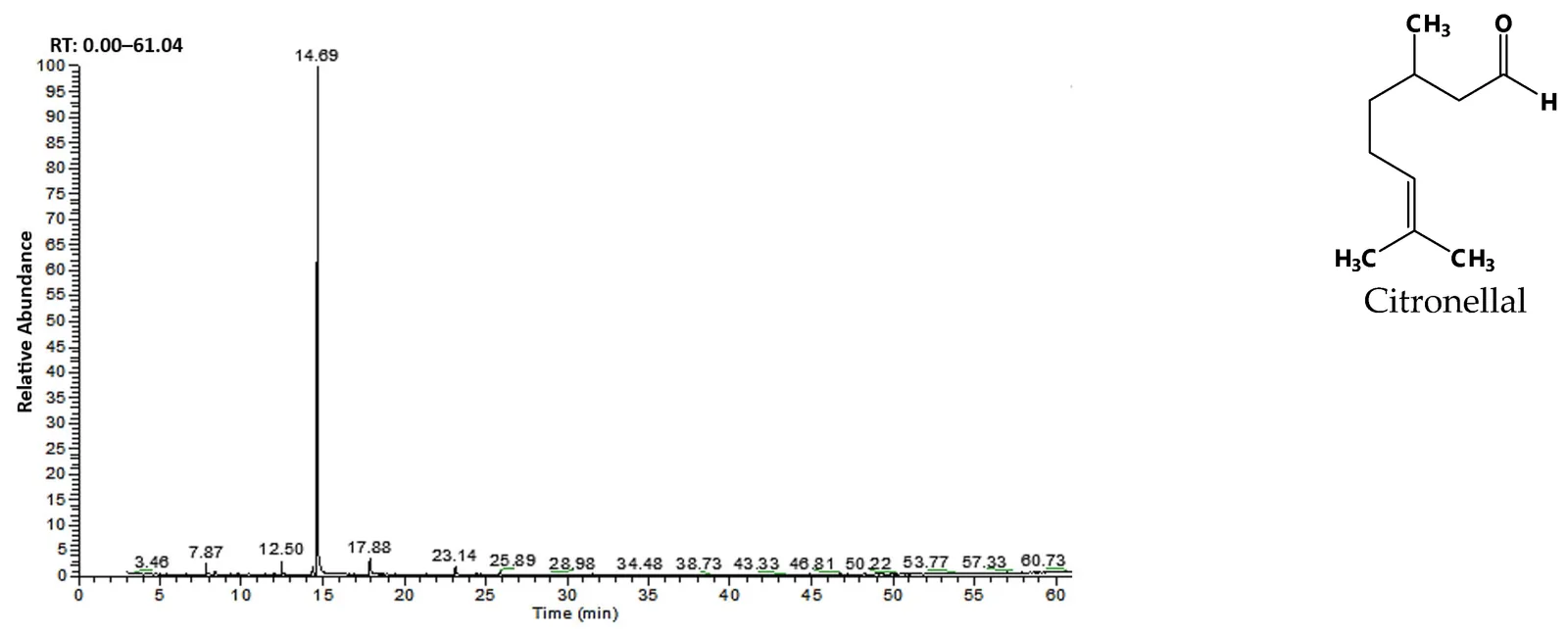
GC-MS chromatograms of essential oil extracted from kaffir lime leaf.

**Figure 3:**
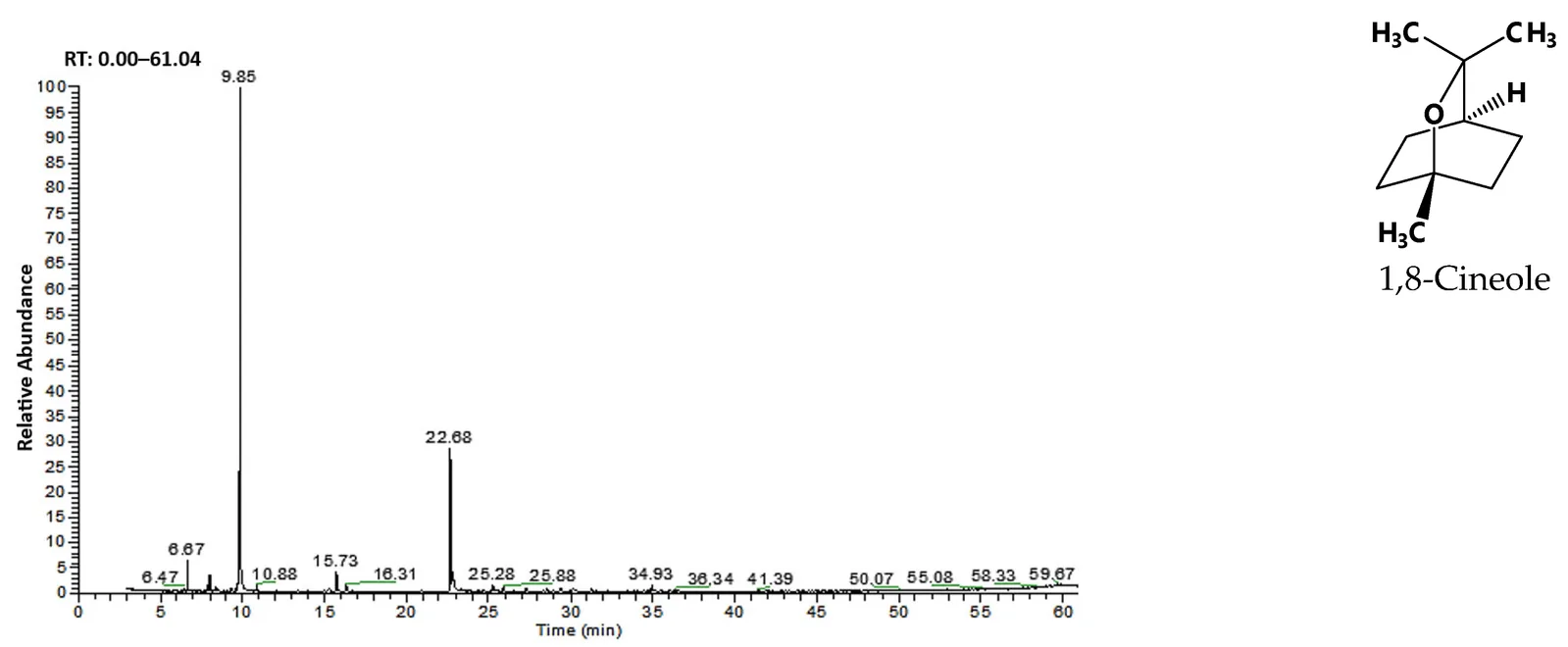
GC-MS chromatograms of essential oil extracted from galangal rhizome.

The chemical compositions of the essential oils were analyzed by gas chromatography–mass spectrometry (GC–MS). The identified compounds and their corresponding relative abundances are listed in Table 2, whereas the GC–MS chromatograms are provided in Figure 1, Figure 2 and Figure 3.

As illustrated in Figure 1, the ginger rhizome essential oil contained four principal constituents: camphene (11.89%; retention time (RT), 7.10 min), 1,8-cineole (13.37%; retention time, 9.85 min), cis-carveol (16.09%; retention time, 18.42 min), and geranial (21.17%; retention time, 19.67 min). Citronellal was the predominant constituent of kaffir lime leaf essential oil, representing 86.89% of the total oil and eluting at 14.69 min (Figure 2). In comparison, galangal rhizome essential oil was mainly composed of 1,8-cineole, which accounted for 57.46% of the oil and had a retention time of 9.85 min (Figure 3).

### 2.3. The Inhibitory Activity of Essential Oils Against Pathogenic Fungi

Based on the MIC values presented in Table 3, the antifungal activities of the three essential oils were compared. The galangal rhizome and kaffir lime leaf essential oils exhibited the greatest activity against *T. interdigitale*, with identical MIC values of 0.125% *v*/*v*. Ginger rhizome essential oil demonstrated the highest activity against *C. albicans*, with an MIC of 0.25% *v*/*v*. In contrast, kaffir lime leaf essential oil was the most effective against *A. niger*, with an MIC of 0.125% *v*/*v*.

Accordingly, two essential oils were selected and combined at ratios of 1:3, 1:1, and 3:1, as shown in Table 4. The resulting formulations were subsequently evaluated for synergistic interaction, no interaction, or antagonistic interaction between the essential oils. This analysis was conducted to identify the combination exhibiting the greatest synergistic antifungal activity, which was then selected for formulation development.

The antifungal activities of the essential-oil combinations were evaluated based on their minimum inhibitory concentration (MIC) and fractional inhibitory concentration index (FICI) values, as presented in Table 4 and Table S1a. Against *T. interdigitale*, the ginger rhizome–kaffir lime leaf essential-oil combination at a 3:1 ratio exhibited the greatest antifungal activity, with an MIC of 0.001953125% *v*/*v* and an FICI value of 0.004643047. Because the FICI value was below 0.5, the combination was considered synergistic. Similarly, the ginger rhizome–kaffir lime leaf combination at a 1:3 ratio demonstrated the highest activity against *C. albicans*, with an MIC of 0.0078125% *v*/*v* and an FICI value of 0.013671875, indicating a synergistic interaction between the two essential oils. Against *A. niger*, the galangal rhizome–ginger rhizome combination at a 1:1 ratio showed the strongest antifungal activity, yielding an MIC of 0.0625% *v*/*v* and an FICI value of 0.15625. This FICI value, which was also below 0.5, indicated a synergistic antifungal effect.

**Figure 4:**
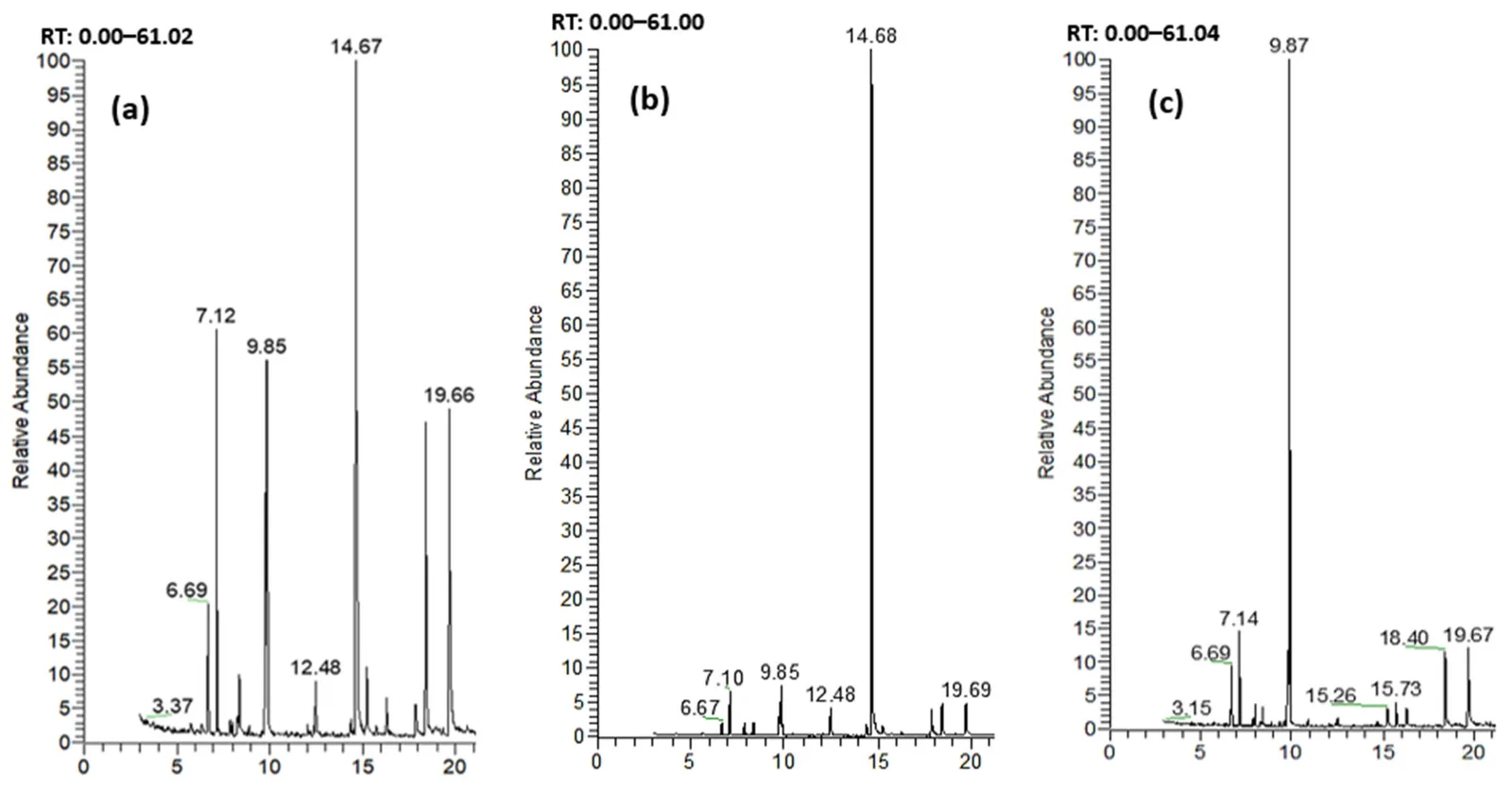
GC-MS chromatograms of blended (**a**) ginger–kaffir lime (3:1); (**b**) ginger–kaffir lime (1:3); (**c**) galangal–ginger (1:1) essential oils.

The antifungal properties of these essential oils are related to both the dominant compounds and the interactions among minor constituents. Ginger oil, with its relatively complex chemical profile, demonstrated the strongest individual activity against *C. albicans*. Kaffir lime oil, dominated by citronellal, was particularly effective against *A. niger* and also active against *T. interdigitale*. Galangal oil, characterized mainly by 1,8-cineole, showed notable activity against *T. interdigitale*. GC-MS chromatograms of combined essential oils, ginger–kaffir lime (3:1), ginger–kaffir lime (1:3), and galangal–ginger (1:1) were shown in Figure 4. The major components found in the combined ginger–kaffir lime (3:1) essential oils were citronellal (retention time 14.68 min), camphene (retention time 7.10 min), 1,8-cineole (retention time 9.85 min), cis-carveol (retention time 18.42 min), and geranial (retention time 19.67 min), while citronellal was also the predominant component in the ginger–kaffir lime (1:3) essential oil mixture. Camphene, 1,8-cineole (major component), cis-carveol, and geranial were found in combined galangal–ginger (1:1) essential oils, suggesting that combined essential oils provided the mixture of components found in those single essential oils. More importantly, blending the oils produced species-specific synergistic effects and reduced the concentrations required to inhibit fungal growth. These findings support the use of selected essential-oil combinations as potentially more effective antifungal preparations than individual oils. However, the optimal blend ratio must be tailored to the target microorganism, since the most effective combination differed among *T. interdigitale*, *C. albicans*, and *A. niger*.

### 2.4. Antifungal Activities of Essential Oils in Oil-Based Formulations

The blended essential oil demonstrating the strongest antifungal activity against each fungal species was selected as the active ingredient for the preliminary development of oil-based formulations. Three carrier oils—cold-pressed coconut oil, olive oil, and mineral oil—were evaluated as solvents to determine the formulation and concentration with the greatest antifungal efficacy.

**Figure 5:**
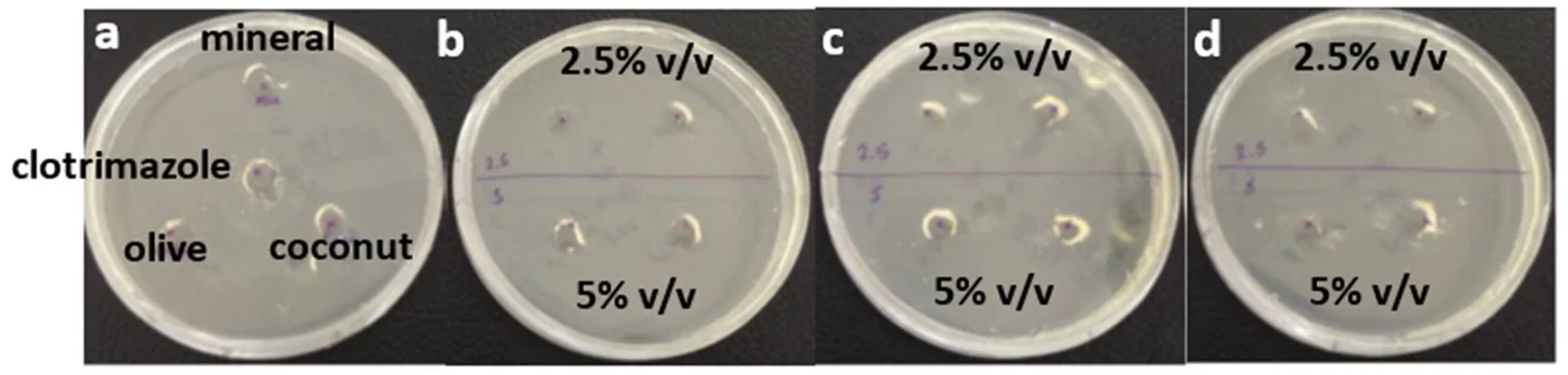
Agar-well diffusion of a 3:1 ginger rhizome–kaffir lime leaf essential oil blend at concentrations of 2.5% and 5% in various carrier oils against *T. interdigitale*: (**a**) controls; (**b**) mineral oil; (**c**) olive oil; (**d**) coconut oil.

**Figure 6:**
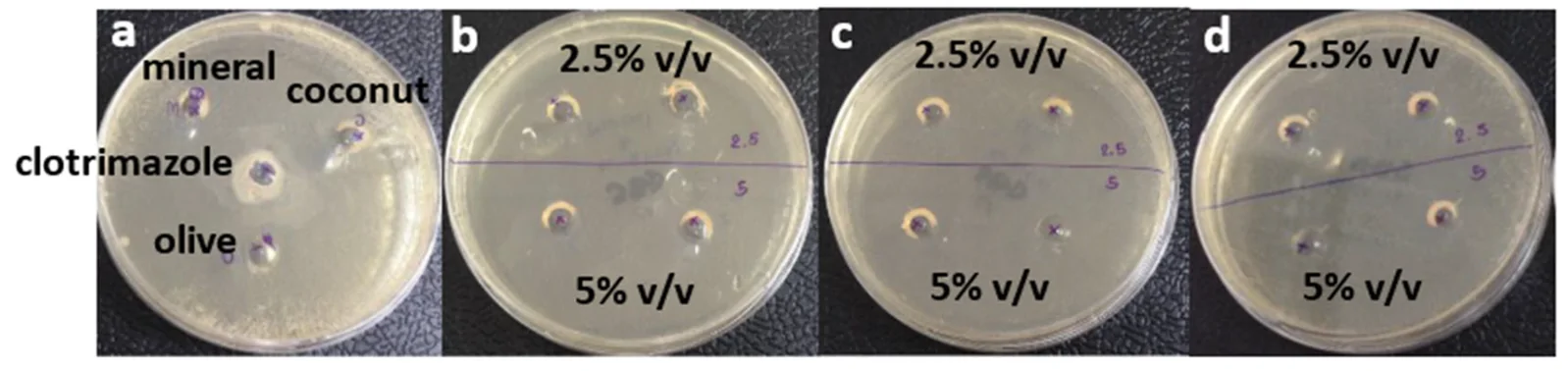
Agar-well diffusion of a 1:3 ginger rhizome–kaffir lime leaf essential oil blend at concentrations of 2.5% and 5% in various carrier oils against *C. albicans*: (**a**) controls; (**b**) mineral oil; (**c**) olive oil; (**d**) coconut oil.

**Figure 7:**
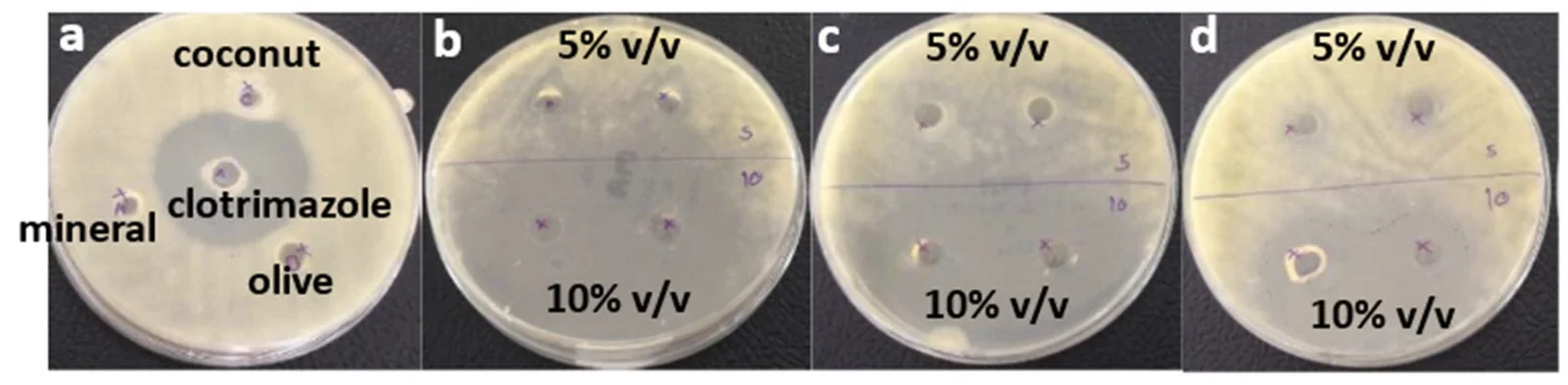
Agar-well diffusion of a 1:1 ginger rhizome–galangal rhizome essential oil blend at concentrations of 5% and 10% in various carrier oils against *A. niger*: (**a**) controls; (**b**) mineral oil; (**c**) olive oil; (**d**) coconut oil.

As shown in Table 5 and Table S1b, the ginger rhizome–kaffir lime leaf essential-oil blend at a 3:1 ratio was evaluated against *T. interdigitale* at concentrations of 2.5% and 5% *v*/*v* using the agar well diffusion method. Both concentrations produced inhibition zones greater than 6 cm in formulations containing all three carrier oils; however, all the carrier oils and 10 mg/mL clotrimazole produced inhibition zones greater than 6 cm too; hence, it cannot compare the antifungal activity of the blended essential oils in oil-based formulations against *T. interdigitale* (Figure 5). Against *C. albicans*, the ginger rhizome–kaffir lime leaf blend at a 1:3 ratio produced inhibition zones greater than 6 cm at both 2.5% and 5% *v*/*v* when mineral oil was used as the carrier, while the mineral oil itself produced no inhibition zone. No inhibition zone was observed in the formulations containing coconut oil and its base oil (Figure 6). In the olive-oil formulations, no inhibition zone was detected at 2.5% *v*/*v*; however, a 3.50 ± 0.50 cm inhibition zone was observed at 5% *v*/*v*, while olive oil showed no inhibition zone, and 10 mg/mL clotrimazole exhibited an inhibition zone of 6 cm (Figure 6). For *A. niger*, the ginger rhizome–galangal rhizome blend at a 1:1 ratio produced no inhibition zone at 2.5% *v*/*v* in formulations containing any of the three carrier oils. Hence, we formulated 5% *v*/*v* and 10% *v*/*v* to evaluate their antifungal activity against *A. niger*. The 5% *v*/*v* mineral-oil formulation exhibited an inhibition zone of 2.15 ± 1.22 cm, the olive-oil formulation showed an inhibition zone of 0.63 ± 0.55 cm, while the coconut-oil formulation possessed no inhibition zone. At 10% *v*/*v*, an inhibition zone greater than 6 cm was observed in the mineral-oil formulation, while inhibition zones of 3.10 ± 0.62 cm and 3.58 ± 0.48 cm were detected in the coconut-oil formulation and the olive-oil formulation, respectively. All base oils presented no inhibition zone, while 10 mg/mL clotrimazole expressed an inhibition zone of 3 cm (Figure 7).

This preliminary antifungal activity of blended essential oils in oil-based formulations study suggested that mineral oil was the most effective carrier for expressing the antifungal activity of the blended essential oils, particularly against *C. albicans* and *A. niger*. Coconut oil was the least suitable carrier under the tested conditions, while olive oil produced intermediate activity. The results demonstrate that carrier selection is a critical factor in essential-oil formulation development. However, when an essential-oil blend has a strong synergistic activity, an unsuitable carrier can reduce the availability of its active constituents and substantially weaken the apparent antifungal effect. Therefore, the mineral-oil formulations should be prioritized for further development, with additional evaluation of their physical stability, active-compound release, skin compatibility, skin irritation, and antifungal performance under conditions that more closely represent practical use. Clinically isolated fungal strains should be used as models for evaluating the potential of these formulations as topical antifungal agents.

## 3. Discussion

The antifungal activity of the essential oils was closely related to their chemical composition, particularly the abundance of oxygenated monoterpenes and aldehydes [17,18]. Ginger oil contained geranial (21.17%), cis-carveol (16.09%), 1,8-cineole (13.37%), and camphene (11.89%). The presence of 1,8-cineole (eucalyptol) and camphene was also reported by Kamal et al. (2023) in essential oil from fresh ginger cultivars grown in Thailand [19] and in ginger oil from Romania [20]. Its chemically diverse profile may explain its broad activity, as multiple constituents can act simultaneously on fungal membranes, membrane-associated enzymes, and metabolic pathways. Oxygenated compounds may increase membrane permeability and disrupt cellular function [21], which may account for ginger oil’s greatest activity against *C. albicans* (MIC 0.25% *v*/*v*).

Kaffir lime oil was dominated by citronellal (86.89%), which is consistent with the reports of chemical compositions of kaffir lime oil from Thailand cultivars [14,16] and from Vietnam cultivars [22]. This aldehydic compound may interact with membrane components and cellular proteins [23,24], contributing to the strong activity observed against *T. interdigitale* and *A. niger* (MIC 0.125% *v*/*v*). Nevertheless, the activity should not be attributed solely to citronellal because minor constituents may affect oil stability, penetration, and efficacy through additive or synergistic interactions [25]. Galangal oil contained a high proportion of 1,8-cineole (57.46%), which is consistent with those reports from Thailand cultivar [15], Indonesia [26], and India [27], which may disrupt fungal membranes and interfere with cellular respiration [28,29,30]. Its strong activity against *T. interdigitale* (MIC 0.125% *v*/*v*) demonstrates that chemically different oils can produce comparable effects through different dominant constituents.

The fungi showed distinct susceptibility patterns. *T. interdigitale* was most sensitive to galangal and kaffir lime oils, *C. albicans* to ginger oil, and *A. niger* to kaffir lime oil. These differences may reflect variations in cell-wall structure [31,32], membrane lipid composition, growth form, enzyme systems, and tolerance to hydrophobic compounds. Thus, essential-oil activity is both composition- and organism-dependent.

Combining the oils substantially enhanced antifungal activity. Against *T. interdigitale*, the ginger–kaffir lime blend at a 3:1 ratio produced the strongest effect (MIC 0.001953125% *v*/*v*; FICI 0.004643047), indicating pronounced synergy. Ginger constituents may have contributed primary antifungal activity, while citronellal may have enhanced membrane disruption and compound penetration. Against *C. albicans*, the optimal blend was ginger–kaffir lime at 1:3 (MIC 0.0078125% *v*/*v*; FICI 0.013671875), suggesting that the higher citronellal content was particularly beneficial for this yeast. Against *A. niger*, the galangal–ginger blend at 1:1 showed synergistic activity (MIC 0.0625% *v*/*v*; FIC 0.15615), although its weaker effect compared with the other combinations may reflect the greater resistance of this mold. However, we studied only three combination ratios (1:3, 1:1, and 3:1), which did not reflect the real best synergistic ratios; hence, a checkerboard assay should be performed to obtain the best synergistic ratio.

The synergistic antifungal activity of ginger, kaffir lime, and galangal essential oils (EOs) against *T. interdigitale*, *A. niger*, and *C. albicans* may be attributed to complementary, multitarget effects, particularly those involving the fungal cell membrane.

First, lipophilic EO constituents may disrupt membrane integrity and perturb ergosterol-rich domains, increasing membrane permeability and impairing proton-gradient-dependent processes. These changes may facilitate the entry or enhance the activity of other EO constituents or co-administered antifungal agents [33,34,35,36]. Second, compounds such as phenylpropanoids may affect intracellular processes, including energy metabolism, oxidative balance, and enzyme activity. When combined with constituents acting on other targets, these effects may impose broader antifungal pressure on the cell [36,37]. Third, at subinhibitory concentrations, some EO constituents may reduce *C. albicans* germ-tube formation and biofilm development. Such effects could complement the growth-inhibitory activity of other oils or antifungal agents, although their contribution to therapeutic outcomes requires further investigation [35,37,38]. Finally, formulation characteristics may influence the magnitude of these interactions. Emulsions and nanocarriers can improve dispersion and contact between hydrophobic EO constituents and fungal cells, potentially enhancing local delivery and activity in topical applications, including those targeting dermatophytes such as *T. interdigitale* [14,35,39].

These mechanisms provide a plausible framework for understanding EO synergy; however, direct experimental evidence is needed to determine which processes account for the observed effects in each fungal species.

The vapor phase of many essential oils, for example, Kaffir lime [40], mustard [41], and lemongrass [42], possesses antimicrobial activity; therefore, the vapor of essential oil might interfere with the inhibition zone when performing an agar diffusion assay, giving less microbial growth observed in the culture plate. In a broth micro-dilution assay performed in a 96-well plate, the vapor phase can interfere with the growth of microbes in the plate, leading to less or no microbial growth observed. Hence, we performed the control plate for the agar diffusion assay in a different plate to prevent this possible effect. However, we observed that 10 mg/mL clotrimazole interfered with the growth of *T. interdigitale* and *C. albicans* by exhibiting an inhibition zone greater than 6 cm (against *T. interdigitale*), covering the base-oils and completely inhibiting the growth of *T. interdigitale* in the control plate (Figure 5a, Table 5), and exhibiting an inhibition zone of 6 cm (against *C. albicans*), almost completely inhibiting the growth of *C. albicans* in the control plate (Figure 6a, Table 5). In future studies, the concentration of clotrimazole as a positive control for *T. interdigitale* and *C. albicans* should be reduced to prevent overgrowth inhibition, while 10 mg/mL clotrimazole can be used as a positive control against *A. niger* (inhibition zone at 3 cm) (Figure 7a, Table 5).

Carrier oil strongly influenced the performance of the formulations. Mineral oil was the most effective carrier, whereas coconut oil generally produced the weakest activity. The ginger–kaffir lime blend was highly active against *T. interdigitale* in all carriers, indicating strong intrinsic synergy. However, against *C. albicans* and *A. niger*, only mineral-oil formulations produced substantial inhibition zones, particularly at 5% *v*/*v* for *C. albicans* and 10% *v*/*v* for *A. niger*. Olive oil showed intermediate activity, while coconut oil produced little or no inhibition. These differences may result from variations in viscosity, polarity, lipid composition, compound retention, and diffusion through agar. Mineral oil may provide a relatively inert, nonpolar medium that maintains essential-oil availability and permits gradual release. In contrast, triglycerides and minor constituents in vegetable oils may retain or alter volatile compounds and limit their diffusion [43].

In our study, mineral oil was the most effective carrier for expressing the antifungal activity of the blended essential oils, particularly against *C. albicans* and *A. niger*. Coconut oil was the least effective carrier under the tested conditions, whereas olive oil produced intermediate activity. These differences may reflect carrier-dependent effects on the diffusion and availability of essential-oil constituents in the assay, rather than differences in the intrinsic antifungal potency of the blend. Because diffusion-based assays can be influenced by the carrier’s physical properties, the results should be interpreted as apparent antifungal activity under the test conditions and do not, by themselves, establish differences in therapeutic efficacy or delivery.

Carrier oils differ in viscosity and physical state, and these properties can vary with temperature and oil composition. Mineral oil is generally less viscous than vegetable oils, although its viscosity depends on grade. Coconut oil is particularly temperature-sensitive: it may solidify or become semisolid at lower temperatures and liquefy as the temperature rises. Olive oil remains liquid under typical assay conditions but is generally more viscous than light mineral oil.

These differences may influence the spreading and diffusion of essential-oil constituents through agar, thereby affecting the apparent antifungal activity measured in a diffusion assay. Under the conditions used in this study, mineral oil may have spread or allowed release of the essential-oil constituents more readily than olive oil and coconut oil, respectively. This could partly explain the stronger inhibition observed with the mineral-oil formulation. However, viscosity alone cannot be assumed to account for the differences: diffusion may also depend on assay temperature, carrier composition, essential-oil solubility and release, and interactions with the agar. Accordingly, these results indicate carrier-dependent differences in apparent activity in the assay, rather than directly demonstrating differences in therapeutic efficacy.

Nevertheless, the findings indicate that carrier selection is an important consideration in essential-oil formulation development. A carrier that limits the release or diffusion of active constituents may reduce the observed inhibitory effect, even when the essential-oil blend is active. Mineral-oil formulations therefore warrant further investigation, including assessment of physical stability, active-compound release, skin compatibility, and antifungal performance in tests that better represent practical use.

The findings should be interpreted cautiously because agar diffusion measures both antifungal activity and formulation-dependent migration. A small or absent inhibition zone may therefore reflect poor release rather than inadequate antifungal potency. Future research should confirm these results using broth microdilution, time-kill assays, and checkerboard analyses, while chemical profiling before and after formulation could clarify compound stability and synergistic interactions. Various clinically isolated strains of the fungi should be tested to confirm the practical use of the blended oils and blended oils in oil-based formulations. Release studies, viscosity measurements, and particle-size or emulsion characterization would help explain carrier effects. Further work should also assess cytotoxicity, skin compatibility, and long-term stability. Finally, in vivo studies are needed to determine whether the most effective blends, particularly ginger–kaffir lime and galangal–ginger formulations in mineral oil, are safe and effective under practical application conditions.

## 4. Materials and Methods

### 4.1. Plant Materials

Ginger rhizome (*Zingiber officinale* Roscoe), galangal rhizome (*Alpinia galanga* (L.) Willd.), and kaffir lime leaves (*Citrus hystrix* DC.) were collected from the Botanical Garden at the Faculty of Pharmacy, Srinakharinwirot University, Nakhon Nayok Province, Thailand, during the months of July and August 2015. The plant materials were identified by Associate Professor Dr. Sarin Tadtong. Voucher specimens (No. SWU_ZO_2026_001 (ginger); SWU_AG_2026_001 (galangal); SWU_CH_2026_001 (kaffir lime)) were deposited in the herbarium of the Faculty of Pharmacy, Srinakharinwirot University, Thailand.

### 4.2. Extraction of Essential Oil

The plant material (ginger rhizome 2000 g, galangal rhizome 2000 g, and 300 g of kaffir lime leaf) was cut into small pieces and then subjected to hydrodistillation using a 5 liter-Clevenger apparatus. Galangal and ginger rhizomes were separated into 3 batches due to limitations of our equipment, then hydrodistilled, and each distilled-batch oil was collected together in an amber bottle. The extracted volatile oil was collected and stored at 2–4 °C until use [14,16].

### 4.3. Essential Oil Composition Analysis

Chemical analysis was performed by gas chromatography–mass spectrometry (GC/MS) using a Finnigan Trace GC Ultra system (Thermo Electron Corporation, Waltham, MA, USA) coupled with a quadrupole mass spectrometer [16]. Separation was achieved on a ZB-5 fused-silica capillary column (30 m × 0.22 mm internal diameter; film thickness, 0.25 μm). The oven temperature was programmed from 50 to 250 °C at a rate of 7 °C/min. The injector and detector temperatures were maintained at 250 and 280 °C, respectively. A 1 μL sample was injected at a split ratio of 100:1, with helium serving as the carrier gas at a flow rate of 2 mL/min. Compound identification was based on comparisons of the Kovats gas chromatographic retention indices obtained using the HP-5MS column with published literature values, computer-assisted matching through the MassLynx database, and evaluation of mass-spectral fragmentation patterns against previously reported data [44].

### 4.4. Antifungal Assays Using Agar Diffusion and Broth Microdilution Methods

The inhibition of *Candida albicans* ATCC10231, *Trichophyton interdigitale* ATCC9533 (formerly *Trichophyton mentagrophytes* var. *interdigitale*), and *Aspergillus niger* ATCC10578 were assessed utilizing the agar diffusion method and the broth microdilution method, employing Sabouraud’s dextrose medium, Potato dextrose medium, and Malt extract medium as the growth promotion media for *C. albicans*, *T. interdigitale*, and *A. niger*, respectively. The preparation procedures are outlined as follows:

#### 4.4.1. Culturing Yeast and Fungi

*Candida albicans* ATCC10231 was cultured in 5 mL Sabouraud’s dextrose broth (Bacton, Dickinson and Company, Sparks, MD, USA) [45] and incubated at 28 °C for 24 h, while *Trichophyton interdigitale* ATCC9533 was cultured in 5 mL potato dextrose broth (Bacton, Dickinson and Company, Sparks, MD, USA) [46] and incubated at 28 °C for 7 days. *Aspergillus niger* ATCC10578 was incubated at 28 °C for 24 h in 5 mL malt extract broth [47]. All microorganisms were obtained from American Type Culture Collection, Manassas, VA, USA.

#### 4.4.2. Preparation of Malt Extract Medium

To prepare 100 mL of Malt extract agar, as recommended by ATCC [48], firstly, 2 g of malt extract (Bacton, Dickinson and Company, Sparks, MD, USA), 2 g of glucose (Unilab, Seven Hills, NSW, Australia), and 0.1 g of peptone (Bacton, Dickinson and Company, Sparks, MD, USA) were dissolved in 100 mL of distilled water. Next, add 2 g of agar (HiMedia, Thane, Maharashtra, India) to the mixture and ensure thorough mixing. Autoclave the suspension at 121 °C to achieve sterilization, and after autoclaving, allow it to cool. Finally, pour the mixture into sterile 90 mm Petri plates within a Class II Biohazard Safety Cabinet (ESCO Lifesciences, Singapore) and permit the agar to solidify before use in experiments. Malt extract broth was prepared as described above without adding agar.

#### 4.4.3. Antifungal via Broth Microdilution Method

To determine the minimum inhibitory concentration (MIC) of the microorganisms using the broth microdilution method, first prepare a 2% DMSO (Merck, Darmstadt, Germany) solution in appropriate medium (as described in Section 4.4.1). Adjust the fungus or yeast culture to a density corresponding to McFarland No. 0.5 and dilute this suspension to a concentration of 5% in the broth (final concentration was 2.5% *v*/*v*). The filamentous fungi were cultured in 5-ml broth, and the fungal colony was transferred into 10-ml sterile water, then dissociated into small pieces by sonication for 2–3 min. The visualized fragments of colonies and filaments were adjusted to a turbidity equal to McFarland No. 0.5 before use. Essential oils were diluted with 2% DMSO in medium to make 2-fold serial dilution concentrations (final concentrations 0.00097765625-4% *v*/*v* in 1%DMSO in medium). Next, pipette 100 μL of the diluted microbial suspension and the various concentrations of essential oils 100 μL into a 96-well plate designed for the broth microdilution method (three wells for each concentration). The experiment was performed for three independent experimental repeats (*n* = 3, triplicate). The plates were incubated at 28 °C for 24 h for *C. albicans*, 72 h for *A. niger*, and 7 days for *T. interdigitale*. Subsequently, the lowest concentration at which there was no observable growth in all replicates from the three experiments was determined as the MIC [14,16]. Medium was used as a negative control, representing fungal growth without an inhibitor. A 2% DMSO solution in the broth (final concentration of 1% *v*/*v*) was used as a solvent control, representing fungal growth without interference from the solvent. Clotrimazole (Sigma-Aldrich, St. Louis, MO, USA) at a final concentration of 500 µg/mL in the medium was used as a positive control, representing complete inhibition of fungal growth.

#### 4.4.4. Evaluation of the Synergistic Effects of Combined Essential Oils Using the Broth Microdilution Method

The combined essential oil was prepared by mixing ginger rhizome–kaffir lime leaf oils, galangal rhizome–kaffir lime leaf oils, and ginger rhizome–galangal rhizome oils in ratios of 1:1, 1:3, and 3:1. The minimum inhibitory concentration (MIC) of the mixed essential oils was then determined using the method described in Section 4.4.3.

The mixed essential oils were analyzed to assess whether they exhibited synergistic, no interaction, or antagonistic effects by calculating the Fractional Inhibitory Concentration Index (FICI) [49]. The interpretation of FICI is as follows:

If FICI > 4, it indicates an antagonistic effect of the mixed essential oils.

If FICI > 0.5 to ≤ 4, it signifies no interaction of the mixed essential oils.

If FICI ≤ 0.5, it demonstrates a synergistic effect of the mixed essential oils.

The FICI can be calculated using the following formula:

$$FICI=\frac{a}{MICA}+\frac{b}{MICB}$$

where

MICA is the MIC of essential oil A (first essential oil).
MICB is the MIC of essential oil B (second essential oil).
a represents the proportion of essential oil A multiplied by the MIC of the mixed essential oils.
b represents the proportion of essential oil B multiplied by the MIC of the mixed essential oils.

#### 4.4.5. Antifungal Activity of Essential Oils in Oil-Based Formulations via Agar-Well Diffusion Method

The antifungal assay using the agar-well diffusion method was performed by making wells in an agar plate using a sterile cylinder cup with a diameter of 6 mm. The appropriate agar medium (as described in Section 4.4.1) was then inoculated with fungus or yeast using a sterile swab dipped in a culture adjusted to a turbidity equivalent to McFarland standard No. 0.5. The swab was gently dried against the side of the test tube before swabbing the agar plate surface, which was then allowed to stand for 3–5 min. Prepare a positive control by dissolving clotrimazole in methanol to achieve a final concentration of 10 mg/mL. Using a micropipette (Gilson, Villiers-le-Bel, France), 100 µL of the prepared essential oil formulation and oil base was deposited into the prepared agar wells, with two wells per plate spaced appropriately. The experiment was repeated three times (*n* = 3, duplicate). Apply 100 µL of 10 mg/mL clotrimazole into an agar well as a positive control, repeating this procedure three times. Additionally, apply 100 µL of oil base into an agar well as a negative control, repeating this step three times. The plates were incubated at 28 °C for 24 h for *C. albicans*, 72 h for *A. niger*, and 7 days for *T. interdigitale*, after which the diameter of the inhibition zone where fungal or yeast growth was absent was measured [14,16].

### 4.5. Statistical Analysis

Statistical analysis of the agar-well diffusion assay was performed using analysis of variance (one-way ANOVA), followed by Tukey post hoc in OriginPro 9.0. Data are expressed as mean ± standard deviation (SD) derived from three independent experimental replicates. Differences were considered significant only when the *p*-value was less than 0.05.

## 5. Conclusions

The antifungal activity of ginger, kaffir lime, and galangal essential oils was strongly associated with their major chemical constituents and varied according to the fungal species. Ginger oil was most effective against *C. albicans*, whereas kaffir lime and galangal oils showed greater activity against *T. interdigitale* and *A. niger*. Synergistic interactions were greatest in ginger–kaffir lime combinations against *T. interdigitale* and *C. albicans*, while galangal–ginger showed moderate synergy against *A. niger*. Mineral oil enhanced formulation performance, highlighting the importance of carrier selection. Further studies on checkerboard assay for evaluating the best blended synergistic antifungal essential oil, mechanism of action of the blended oils, antifungal activity against various clinically isolated fungal strains, standardization, toxicology, stability, in vivo studies, and clinical studies of the blended oils in oil-based formulation are required.

## 6. Patents

The antifungal blended essential oil formulas against *T. interdigitale* (ginger–kaffir lime blend at a 3:1 ratio, Thailand Petty Patent 14491) [50], *C. albicans* (ginger–kaffir lime blend at a 1:3 ratio, Thailand Petty Patent 14489) [51], and *A. niger* (ginger–galangal blend at a 1:1 ratio, Thailand Petty Patent 14490) [52] were reported in this manuscript.

## Figures and Tables

**Table 1:** Hydrodistillation extraction yields of essential oils from ginger rhizome, kaffir lime leaf, and galangal rhizome.

Plant Samples	Ginger Rhizome	Kaffir Lime Leaf	Galangal Rhizome
Yield (%*w*/*w*)	0.09	0.37	0.16

**Table 2:** Chemical composition of essential oils from ginger rhizome, kaffir lime leaf, and galangal rhizome.

RT	Compounds	Kovat’s Index	Area%		
			Ginger Rhizome	Kaffir Lime Leaf	Galangal Rhizome
5.67	Heptanol<2->	931	1.07		
6.47	Thujene<alpha->	930			0.31
6.67	Pinene<alpha->	939	3.23		2.47
7.10	Camphene	954	11.89		
7.87	Sabinene	975		1.55	0.67
7.97	Pinene<beta->	979	0.46		1.82
8.24	Hepten-2-one<6-methyl-5->	985	1.56		
8.38	Myrcene	990	1.99	0.57	0.60
8.88	Phellandrene<alpha->	1002	0.45		
9.31	Terpinene<alpha->	1017			0.37
9.77	Limonene	1029	9.42		1.59
9.85	Cineole<1,8->	1031	13.37		57.46
10.45	Ocimene<(Z)-beta->	1037		0.19	
10.88	Terpinene<gamma->	1059			0.61
11.43	Linalool oxide <cis->	1072		0.28	
12.04	Unidentified			0.22	
12.38	Unidentified		0.40		
12.48	Linalool	1096	1.66	2.41	
14.35	Camphor	1146	0.52		
14.40	Isopulegol	1149		1.42	
14.69	Citronellal	1153	0.91	86.89	
15.26	Borneol	1169	3.10		
15.29	Terpineol<delta->	1166			0.61
15.75	Terpinen-4-ol	1177	0.42	0.24	2.74
16.31	Terpineol<alpha->	1188	1.87		1.09
17.91	Citronellol	1225	0.77	4.16	
18.42	Carveol<cis->	1229	16.09		
18.94	Unidentified		0.54		
19.67	Geranial	1267	21.17		
20.67	Undecanone<2->	1294	0.39		
20.92	Unidentified				0.35
22.68	Fenchyl acetate<alpha>	1347			18.50
22.80	Chavicol	1350			4.31
23.14	Citronellyl acetate	1352		1.47	
23.34	Carvyl acetate<cis->	1367			0.35
25.28	Methyl eugenol	1403			1.19
25.89	Caryophyllene(E-)	1419		0.60	0.65
27.25	Humulene<alpha->	1454			0.86
28.44	Curcumene<ar->	1480	1.63		
28.55	Selinene<beta->	1490			0.39
28.93	Selinene<alpha->	1498			0.37
28.94	Zingiberene<alpha->	1493	2.86		
29.45	Farnesene<(E,E)-alpha->/Bisabolene<beta->	1505	1.35		0.53
30.05	Sesquiphellandrene<beta->/Eugenol acetate	1522	1.09		0.68
31.31	Germacrene B	1561			0.45
33.47	Unidentified		0.47		
33.97	Unidentified		0.73		
34.82	Eudesmol<beta->	1650	0.57		
34.93	Eudesmol<gamma->	1632			1.02

**Table 3:** Minimum inhibitory concentrations (MIC; % *v*/*v*) of ginger rhizome, kaffir lime leaf, and galangal rhizome essential oils against pathogenic fungi.

Plant Samples	MIC (% *v*/*v* of Essential Oil)		
	***T. interdigitale***	***C. albicans***	***A. niger***
Ginger rhizome oil	2	0.25	1
Kaffir lime leaf oil	0.125	1	0.125
Galangal rhizome oil	0.125	0.5	0.25

**Table 4:** Synergistic effects of combined essential oils on *T. interdigitale*, *C. albicans* and *A. niger*.

Blended Essential Oils Ratios			MIC (%*v*/*v*)	FICI	Results
Galangal	Kaffir lime	Ginger	*T. interdigitale*		
1	3	0	0.25	2	No interaction
1	1	0	0.0625	0.5	Synergy
3	1	0	0.25	2	No interaction
1	0	3	0.3125	0.7421875	No interaction
1	0	1	0.0625	0.265625	Synergy
3	0	1	0.3125	1.9140625	No interaction
0	3	1	0.015625	0.095555	Synergy
0	1	1	0.0078125	0.033203125	Synergy
0	1	3	0.0019553125	0.004643047	Synergy
			***C. albicans***		
1	3	0	1	1.25	No interaction
1	1	0	0.5	0.75	No interaction
3	1	0	1	1.75	No interaction
1	0	3	0.125	0.4375	Synergy
1	0	1	1	3	No interaction
3	0	1	0.25	0.625	No interaction
0	3	1	0.0078125	0.013671875	Synergy
0	1	1	0.015625	0.0390625	Synergy
0	1	3	0.015625	0.05078125	Synergy
			***A. niger***		
1	3	0	0.125	0.875	No interaction
1	1	0	0.25	2.5	No interaction
3	1	0	0.125	0.625	No interaction
1	0	3	0.125	0.21875	Synergy
1	0	1	0.0625	0.15625	Synergy
3	0	1	0.125	0.40625	Synergy
0	3	1	0.125	0.5625	No interaction
0	1	1	0.125	0.5625	No interaction
0	1	3	0.125	0.34375	Synergy

**Table 5:** Inhibition zones (cm) produced by combined essential oils in oil-based formulations against *T. interdigitale*, *C. albicans*, and *A. niger*.

Carrier Oil	Essential Oil	Inhibition Zone (cm)	
		2.5%	5.0%
*T. interdigitale*			
Coconut oil	Without essential oil	>6	
Olive oil		>6	
Mineral oil		>6	
Clotrimazole 10 mg/mL		>6	
Coconut oil	Ginger–kaffir lime essential-oil blend at a 3:1 ratio	>6	>6
Olive oil		>6	>6
Mineral oil		>6	>6
*C. albicans*			
Coconut oil	Without essential oil	0.00	
Olive oil		0.00	
Mineral oil		0.00	
Clotrimazole 10 mg/mL		6.00	
Coconut oil	Ginger–kaffir lime essential-oil blend at a 1:3 ratio	0.00 ± 0.00 ^b,c,e^	0.00 ± 0.00 ^b,c,e^
Olive oil		0.00 ± 0.00 ^b,c,d^	3.50 ± 0.50 ^a,b,c,d^
Mineral oil		>6 ^a^	>6 ^a^
*A.* *niger*			
		5.0%	10%
Coconut oil	Without essential oil	0.00	
Olive oil		0.00	
Mineral oil		0.00	
Clotrimazole 10 mg/mL		3.00	
Coconut oil	Ginger–galangal essential-oil blend at a 1:1 ratio	0.00 ± 0.00 ^f,g,j^	3.10 ± 0.62 ^a,g,j^
Olive oil		0.63 ± 0.55 ^g,i^	3.58 ± 0.48 ^a,g,i^
Mineral oil		2.15 ± 1.22 ^g,h^	>6 ^a,h^

Remark: Significance considered when a = *p* < 0.05 compared with its base oil; for *C. albicans* when b = *p* < 0.05 compared with 2.5% in mineral oil; c = *p* < 0.05 compared with 5% in mineral oil; d = *p* < 0.05 compared 2.5% in olive oil with 5% in olive oil; e = *p* < 0.05 compared 2.5% in coconut oil with 5% in coconut oil; and for *A. niger* when f = *p* < 0.05 compared with 5% in mineral oil; g = *p* < 0.05 compared with 10% in mineral oil; h = *p* < 0.05 compared 5% in mineral oil with 10% in mineral oil; i = *p* < 0.05 compared 5% in olive oil with 10% in olive oil; and j = *p* < 0.05 compared 5% in coconut oil with 10% in coconut oil.

## Data Availability

The original contributions presented in this study are included in the article. Further inquiries can be directed to the corresponding author.

## Supplementary Materials

The following supporting information can be downloaded at: https://www.mdpi.com/article/10.3390/molecules31213951/s1, Table S1a: FICI calculation of combined essential oils; Table S1b: Statistical analysis of agar-well diffusion assay of combined essential oils in oil-based formulations.

## Abbreviations

The following abbreviations are used in this manuscript:

CFU	Colony-forming unit
DMSO	Dimethyl sulfoxide
FIC	Fractional inhibitory concentration
GC-MS	Gas chromatography-mass spectrometry
HCl	Hydrochloric acid
MIC	Minimum inhibitory concentration
mL	Milliliter
RT	Retention time
i.d.	Internal diameter
ΣFIC	Sum of fractional inhibitory concentrations
°C	Degree Celsius
µg	Microgram
µL	Microliter
µm	Micrometer
